# Value of fecal calprotectin in the evaluation of patients with abdominal discomfort: an observational study

**DOI:** 10.1186/1471-230X-12-5

**Published:** 2012-01-10

**Authors:** Michael Manz, Emanuel Burri, Claude Rothen, Nuschin Tchanguizi, Christian Niederberger, Livio Rossi, Christoph Beglinger, Frank Serge Lehmann

**Affiliations:** 1 Department of Gastroenterology, University Hospital of Basel, Petersgraben 4, 4031 Basel, Switzerland; 2 Digestive Research Unit, University Hospital Vall d'Hebron, Paseo Vall d'Hebron 119-129, 08035 Barcelona, Spain; 3 Rothen Medical Laboratories, Spalengraben 15, 4003 Basel, Switzerland; 4 Bühlmann Laboratories AG, Baselstrasse 55, 4124 Schönenbuch, Switzerland

**Keywords:** Gastroenterology, Endoscopy, Diagnosis, Biomarker

## Abstract

**Background:**

The evaluation of patients with abdominal discomfort is challenging and patient selection for endoscopy based on symptoms is not reliable. We evaluated the diagnostic value of fecal calprotectin in patients with abdominal discomfort.

**Methods:**

In an observational study, 575 consecutive patients with abdominal discomfort referred for endoscopy to the Department of Gastroenterology & Hepatology at the University Hospital Basel in Switzerland, were enrolled in the study. Calprotectin was measured in stool samples collected within 24 hours before the investigation using an enzyme-linked immunosorbent assay. The presence of a clinically significant finding in the gastrointestinal tract was the primary endpoint of the study. Final diagnoses were adjudicated blinded to calprotectin values.

**Results:**

Median calprotectin levels were higher in patients with significant findings (N = 212, median 97 μg/g, IQR 43-185) than in patients without (N = 326, 10 μg/g, IQR 10-23, P < 0.001). The area under the receiver operating characteristics curve (AUC) to identify a significant finding was 0.877 (95% CI, 0.85-0.90). Using 50 μg/g as cut off yielded a sensitivity of 73% and a specificity of 93% with good positive and negative likelihood ratios (10.8 and 0.29, respectively). Fecal calprotectin was useful as a diagnostic parameter both for findings in the upper intestinal tract (AUC 0.730, 0.66-0.79) and for the colon (AUC 0.912, 0.88-0.94) with higher diagnostic precision for the latter (P < 0.001). In patients > 50 years, the diagnostic precision remained unchanged (AUC 0.889 vs. 0.832, P = 0.165).

**Conclusion:**

In patients with abdominal discomfort, fecal calprotectin is a useful non-invasive marker to identify clinically significant findings of the gastrointestinal tract, irrespective of age.

## Background

Abdominal discomfort is a common cause of consultation in primary care and gastroenterology departments alike and presents a clinical challenge even for experienced physicians [1,2]. Unfortunately, patient selection for endoscopy based on symptoms is not reliable [3,4] and a substantial part of patients with abdominal discomfort will suffer from any number of non-organic diseases, e.g. functional gastrointestinal disorders [5,6]. Accordingly, in many patients, endoscopy might not be necessary. The use of diagnostic criteria, including risk factors for organic disease may help to select patients for endoscopy [7,8]. However, the evaluation and risk stratification of this important group of patients with a non-invasive, and widely available test is highly desirable.

Over the past years, fecal calprotectin, a cytosolic protein in neutrophilic granulocytes that correlates well with neutrophilic infiltration of the intestinal mucosa [9], has been investigated as biological marker of intestinal inflammation [10], especially in inflammatory bowel disease (IBD) [11]. Fecal calprotectin reliably distinguished IBD from functional gastrointestinal disorder [12,13] and correlated well with IBD disease activity [14]. Increased levels have also been described in colorectal neoplasia [15], microscopic colitis [16], bacterial diarrhea [17], after the use of non-steroidal anti-inflammatory drugs [18], in peptic ulcer [19], and gastric cancer [20].

The role of fecal calprotectin in unselected patients with abdominal discomfort referred for diagnostic endoscopy has been poorly studied [21]. Specifically, it is not known whether fecal calprotectin can be used as a diagnostic marker of organic gastrointestinal disease. In addition, it should be further explored and validated whether the diagnostic ability of calprotectin in the colon can be expanded to the upper gastrointestinal tract. This has not been investigated before.

The aim of our study was therefore to prospectively investigate the value of fecal calprotectin as a biological marker for the diagnosis of intestinal organic disease in symptomatic patients. To do so, we investigated consecutive patients with abdominal discomfort referred for endoscopy.

## Methods

### Setting and participants

In this observational study, we prospectively investigated patients undergoing endoscopy of the gastrointestinal tract for abdominal discomfort at the Department of Gastroenterology of the University Hospital Basel in Switzerland. Switzerland has an open-access system for endoscopy and the decision to perform endoscopy was based on clinical grounds by the referring physician. Abdominal discomfort was defined as any sensation of any quality and intensity of abdominal pain. If several symptoms were present, abdominal discomfort had to be the main symptom. A total of 575 patients were enrolled in two series of consecutive patients: 405 patients with abdominal discomfort referred for colonoscopy and another 170 patients referred for esophagogastroduodenoscopy (EGD). Patients younger than 18 years old were excluded. The study was carried out according to the principles of the Declaration of Helsinki and the local ethic committees of all participating sites approved the protocol. All patients provided written informed consent before participating in any protocol-specific procedures.

### Endpoint

The presence of a clinically significant finding in the gastrointestinal tract was the primary endpoint. For the purpose of this study, a clinically significant finding was defined by the presence of mucosal inflammation with mucosal breaks.

### Adjudication of the final diagnosis

The final diagnosis was independently adjudicated by two gastroenterologists not involved in patient care, blinded to fecal calprotectin values on the basis of all available medical records pertaining to the individual patient (clinical data, laboratory values, endoscopy report, histology report) according to current recommendations [22-25]. The physicians adjudicated the final diagnosis by choosing one or more diagnoses from a pre-specified list that included the following items: Normal findings, esophagitis LA grade A-D, erosive gastritis/duodenitis, gastric/duodenal ulcer, gastric carcinoma, infectious colitis, crohn's disease, ulcerative colitis, ischemic colitis, microscopic colitis, diverticulitis, adenomatous polyp, hyperplastic polyp, colorectal cancer, other, or unknown. If more than one finding was identified, the degree of mucosal inflammation decided on the final diagnosis. When there was disagreement about the final diagnosis, cases were reviewed and adjudicated in conjunction with a third gastroenterology specialist who was considered an expert in the field.

### Measurement of fecal calprotectin

Calprotectin was measured in a single stool sample in all patients. Patients were instructed to collect the sample at home 24 hours prior to bowel preparation for endoscopy. Samples were delivered on the day of the investigation and stored in a refrigerator before transfer to the study laboratory (Rothen Medical Laboratories, Basel, Switzerland) within 48 hours for analysis. Calprotectin is stable up to seven days at room temperature [26].

Fecal calprotectin was determined using a commercially available enzyme-linked immunosorbent assay (Bühlmann Laboratories AG, Schönenbuch, Switzerland) that measures quantitative calprotectin. Aliquots of approximately 100 mg feces were homogenized in 5 mL extraction buffer. 2 mL of the homogenate was then centrifuged in a microcentrifuge for 5 min at 3000 g and 100 μl of the diluted supernatant (1:50 with incubation buffer) were incubated at room temperature onto a microtiter plate coated with a monoclonal capture antibody highly specific to the calprotectin heterodimeric and polymeric complexes. After incubation, washing, a second incubation with a specific detection antibody, and a further washing step, tetramethylbenzidine (blue color formation) followed by a stop solution (change to yellow color) were added. The absorption was determined at an optical density of 450 nm. The measuring range of the test was 10-600 μg calprotectin/g feces with an intra- and inter-assay coefficient of 4.7% and 4.1%, respectively. The calprotectin cut-off level representing a positive value was equal or greater than 50 μg/g as stated by the manufacturer. All fecal samples were processed within 72 hours after collection. The laboratory personnel carrying out the analysis was blinded to the clinical history and the endoscopic findings of the patients.

### Endoscopy

All patients underwent standard endoscopies performed by 4 senior gastroenterologists who were unaware of fecal calprotectin values at the time of the investigation. All endoscopies were documented on a computer-based datasheet (ViewPoint, GE Healthcare, Chalfont St Giles, U.K.) that included a detailed description of the findings by choosing from a predefined list and electronic storage of all images taken during the investigation. Biopsies were collected if appropriate as decided by the endoscopist. Patients with no significant lesion but elevated fecal calprotectin levels (> 50 μg/g) on initial endoscopy were further investigated with either EGD or colonoscopy. The endoscopists performing the follow up endoscopy were aware of the reason for the investigation (positive test).

### Statistical analysis

Results of numerical data are presented as mean (standard deviation, SD) or median (interquartile range, IQR) where appropriate. The Mann-Whitney U-test (for two independent groups) and the Kruskal-Wallis H-test (for more than two independent groups) were used to compare numerical data and the chi-square test was used to compare categorical data. Correlations between numerical data were determined using Pearson or Kendall's tau correlation coefficient (r) where appropriate. Receiver operating characteristics analyses were carried out to determine the test characteristics of fecal calprotectin to identify a clinically significant finding in the gastrointestinal tract. Test characteristics are presented as sensitivity, specificity, positive and negative likelihood ratios (LR^+^, LR^-^), and positive and negative predictive values (NPV, PPV). Overall accuracy of the test was calculated according to the following formula: (true positive test results + true negative test results)/total population. A p-value smaller than 0.05 was considered to be statistically significant.

The prevalence of a clinically significant gastrointestinal lesion in patients with abdominal discomfort was expected at 35%. We used a nomogram to calculate the sample size [27]: Estimating a sensitivity of 85% and a confidence interval of 95% to detect a clinically significant lesion, the targeted sample size was 500 patients.

## Results

### Patients Characteristics

**Figure 1:**
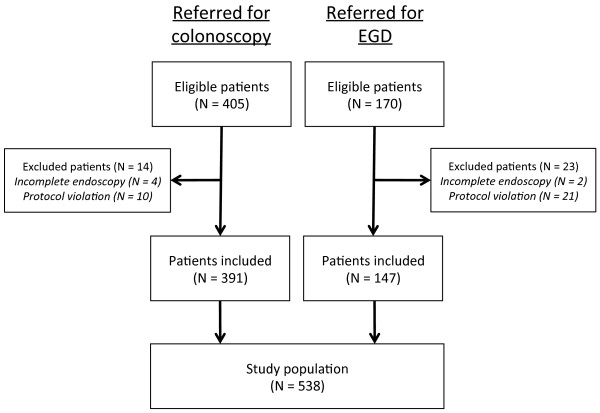
**Study flow**. Study flow of patients referred for colonoscopy and esophagogastroduodenoscopy (EGD). A total of 37 patients were excluded from the final analysis, in 29 patients because follow-up endoscopy was not performed as required by the protocol (no clinically significant lesion on endoscopy but fecal calprotectin > 50 μg/g)

Of 575 patients enrolled in the study, 538 (94%, 248 male, 290 female) were included in the final analysis (Figure 1). Thirty-seven patients (6.4%) were excluded; 6 patients for incomplete endoscopy and 31 patients because they did not complete the diagnostic work up as required by the protocol. Baseline characteristics are shown in Table 1. Overall, 457 colonoscopies (85% of all patients) and 217 EGDs (40%) were performed. Fifteen patients received capsule endoscopy but none revealed significant findings.

**Table 1:** Baseline Characteristics

	Referred for colonoscopy	Referred for EGD	Total
Number of patients	391	147	538
Female patients, N (%)	218 (56%)	72 (49%)	290 (54%)
Age, years	63 (53-71)	55 (42-65)	60 (49-70)
Colonoscopy, N (%)	391 (100%)	66 (45%)	457 (85%)
EGD, N (%)	70 (18%)	147 (100%)	217 (40%)

Data are presented as median (interquartile range) and number of patients (%). EGD: esophagogastroduodenoscopy.

### Clinically significant lesions in patients with abdominal discomfort

Among the study population, the prevalence of a significant finding was 39%. It was higher in patients initially investigated with EGD (47%) than with colonoscopy (37%, P = 0.028). In a majority of patients, endoscopy revealed normal findings (N = 314, 58%). Median calprotectin levels were higher in patients with significant findings (N = 212, median 97 μg/g, IQR 43-185 μg/g) than in patients without (N = 326, 10 μg/g, IQR 10-23, P < 0.001). Table 2 lists adjudicated final diagnoses and respective median values of calprotectin.

**Table 2:** Final diagnoses and fecal calprotectin values

Final diagnosis	N	Median	IQR
**No Clinically Significant Finding**	**326**	**10**	**10 - 23**
Normal findings	314	10	10 - 22
Hyperplastic polyps	12	34	22 - 62
			
**Clinically Significant Finding**	**212**	**97**	**43 - 185**
Esophagitis	31	103	60 - 170
*Esophagitis LA grade A*	*25*	*17*	*11 - 29*
*Esophagitis LA grade B*	*10*	*167*	*59 - 205*
*Esophagitis LA grade C*	*9*	*125*	*62 - 163*
*Esophagitis LA grade D*	*12*	*85*	*66 - 136*
Erosive gastritis/duodenitis	22	70	27 - 156
Gastric ulcers	11	125	66 - 214
Gastric carcinomas	3	355	188 - 881
Colitis/Ileitis	53	152	85 - 338
*Infectious Colitis*	*8*	*127*	*71 - 1317*
*Crohn's disease*	*10*	*69*	*54 - 145*
*Ulcerative colitis*	*16*	*152*	*90 - 281*
*Diverticulitis*	*13*	*260*	*145 - 537*
*Microscopic colitis*	*5*	*281*	*239 - 410*
*Ischemic colitis*	*1*	*314*	*-*
Adenomatous polyps	50	101	25 - 170
Colorectal cancers	17	104	82 - 295

Data are presented as median with interquartile range (IQR).

### Diagnostic value of fecal calprotectin to detect organic disease of the gastrointestinal tract

**Figure 2:**
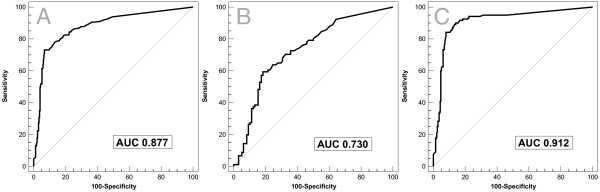
**Receiver Operating Characteristics Curve**. To identify clinically significant findings in the gastrointestinal tract, fecal calprotectin had an area under the receiver operating characteristics (ROC) curve of 0.877 (95% CI 0.85-0.90) (Figure 2A). The AUC of fecal calprotectin to identify clinically significant findings was better in the lower intestinal tract (AUC 0.912, 95%CI 0.88-0.94) (Figure 2C) than in the upper intestinal tract (AUC 0.730, 95%CI 0.66-0-79, P < 0.001) (Figure 2B).

**Figure 3:**
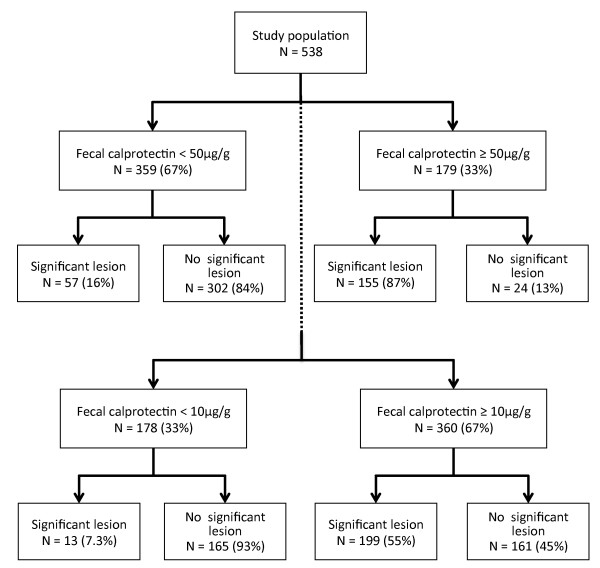
**Diagnostic accuracy of fecal calprotectin**. The overall test accuracy of fecal calprotectin was 85% when 50 μg/g was used as cut off value (upper panel) and was 68% when 10 μg/g was used (lower panel).

Evaluating the value of fecal calprotectin as a diagnostic test to identify significant gastrointestinal findings at endoscopy, we found an area under the receiver operating characteristics curve (AUC) of 0.877 (95% CI, 0.85-0.90, Figure 2A) with an optimal cut-off at 50 μg/g. Using this cut-off yielded a sensitivity of 73% and a specificity of 93% with positive and negative likelihood ratios of 10.8 and 0.29, respectively (Table 3). Fecal calprotectin levels < 10 μg/g indicated that it was very unlikely (sensitivity 94%, LR^+ ^1.9, LR^- ^0.12) to detect a significant lesion at endoscopy, given the high negative predictive value of 93%. The overall accuracy of the test was 85% and 68% using a cut-off of 50 μg/g and 10 μg/g, respectively (Figure 3). Among patients with false negative test results when using 10 μg/g as cut-off were 5 patients with esophagitis LA grade A, 4 patients with colorectal adenoma, and 1 patient with each with colorectal carcinoma of the sigma, gastric ulcer, erosive gastritis, and diverticulitis.

**Table 3:** Test characteristics of fecal calprotectin to predict a clinically significant gastrointestinal finding

Clinically significant finding	AUC (95%CI)	Cut-off (μg/g)	Sens (%)	Spec (%)	LR+	LR-	NPV (%)	PPV (%)	Accuracy (%)
Overall	0.877 (0.85 - 0.90)	50	73	93	10.8	0.29	88	84	85
Upper gastrointestinal tract	0.730 (0.66 - 0.79)	48	59	82	3.2	0.50	75	68	71
Lower gastrointestinal tract	0.912 (0.88 - 0.94)	50	84	92	10.6	0.17	82	93	89

Area under the receiver operating characteristics curve (AUC) with corresponding sensitivity (Sens), specificity (Spec), positive and negative likelihood ratio (LR+, LR-), and negative and positive predictive values (NPV, PPV) for fecal calprotectin to identify a clinically significant finding in the gastrointestinal tract. Overall accuracy was calculated using the following formula: (true positive test results + true negative test results)/total population.

### Fecal calprotectin to identify upper gastrointestinal disease

As a diagnostic test to identify significant findings in the upper gastrointestinal tract, fecal calprotectin showed an area under the receiver operating characteristics curve of 0.730 (95% CI 0.66-0.79). At the optimal cut-off (48 μg/g), fecal calprotectin provided 60% sensitivity and 81% specificity with positive and negative likelihood ratios of 3.23 and 0.50, respectively. The diagnostic ability of fecal calprotectin in the upper intestinal tract was less performant compared to the colon (AUC 0.912, 95% CI 0.88-0.94, P < 0.001, Figure 2, Table 3).

**Figure 4:**
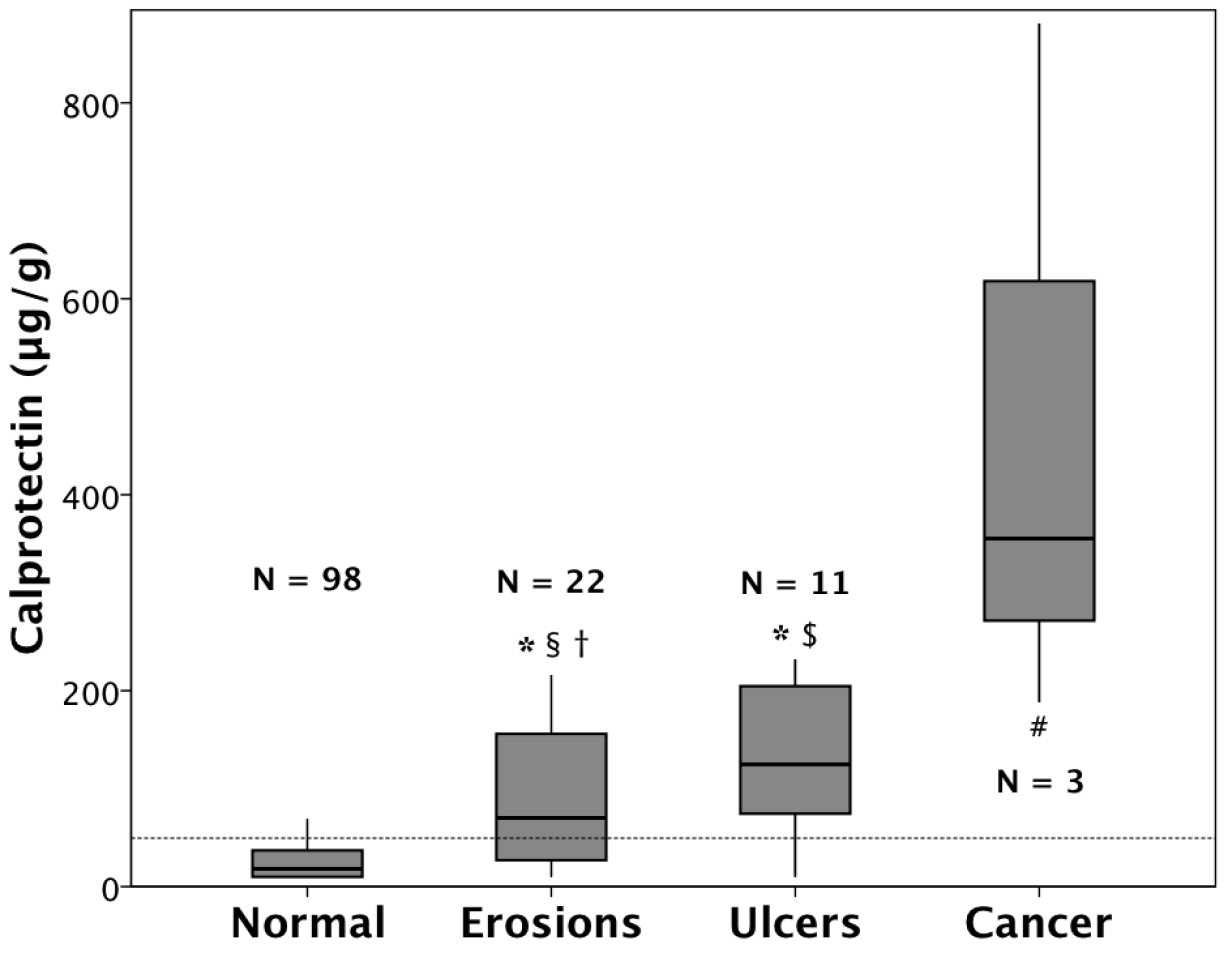
**Fecal calprotectin and severity of mucosal damage**. Boxplots (median, 25^th ^and 75^th ^percentile) of fecal calprotectin values in patients with normal endoscopic findings (normal), gastric erosions (erosion), gastric ulcer (ulcer), and gastric cancer (carcinoma). * P < 0.001 against normal, # P = 0.002 against normal, § P = 0.157 against ulcer, † P = 0.027 against carcinoma, $ P = 0.088 against carcinoma.

Interestingly, in patients with gastric mucosal lesions, fecal calprotectin values increased with disease severity (Figure 4). Values in patients with normal endoscopic findings on EGD (18 μg/g, IQR 10-37) were lower than in patients with erosive gastritis (70 μg/g, IQR 27-156, P < 0.001), peptic ulcers (125 μg/g, IQR 66-125, P < 0.001), and gastric carcinoma (355 μg/g, IQR 188-881, P = 0.002).

### Diagnostic value of calprotectin in patients at higher risk for organic disease

Patients older than 50 years (384 patients, 71%) were not more likely to present a significant finding on endoscopy (42% vs 33%, P = 0.07). When analyzed according to age, the diagnostic ability of fecal calprotectin was similar in patients younger than 50 years (AUC 0.832, 95%CI 0.76-0.89) and in patients older than 50 years (AUC 0.889, 95%CI 0.85-0.92, P = 0.165). The overall accuracy of the test was 83% and 86%, respectively. Among patients with normal findings on endoscopy (314 patients, 58%), older patient groups (divided by decades of age) did not have higher median calprotectin values and no correlation existed between increasing age and calprotectin levels (R = -0.02, P = 0.709).

### Diagnostic value of calprotectin in patients with negative initial endoscopy

One hundred and thirty-six patients (25%) were investigated with both EGD and colonoscopy; 74 patients (54%) had EGD first and 62 (46%) had colonoscopy first. In 38 patients (28%), both procedures were done on the same day for clinical reasons and in 98 patients (82%), follow-up endoscopy was done after no clinically significant findings had been identified during the initial investigation. The median time to follow-up endoscopies was 11 days. Follow-up investigations (51 EGDs and 47 colonoscopies) resulted in 44 additional findings, including 2 colorectal carcinomas, 6 colorectal adenomas, 1 crohn's disease, 1 ulcerative colitis, and 5 peptic ulcers. Patients with significant lesions on follow-up endoscopy had higher median calprotectin levels (139 μg/g (IQR 57-190) vs 40 μg/g (IQR 10-162), P = 0.008) and accordingly, were more likely to have abnormal calprotectin test results (57% vs 43%, P = 0.003). In 10 patients (26%), test results were falsely negative. The overall test accuracy of fecal calprotectin was 63%.

## Discussion

This prospective study in a large cohort of patients examined the use of calprotectin measurement in feces as a diagnostic test to identify clinically significant gastrointestinal findings in patients with abdominal discomfort referred for endoscopy. We provide the following new information: Patients with clinically significant findings at endoscopy had higher fecal calprotectin values than patients without; fecal calprotectin measured before endoscopy reliably predicted the presence of significant findings throughout the gastrointestinal tract; fecal calprotectin provided valuable diagnostic ability for significant findings in the upper intestinal tract but performed less well compared to findings in the colon; the diagnostic performance was independent of age > 50 years, an important risk factor for organic disease; and calprotectin levels indicated disease severity in patients with mucosal lesions of the stomach.

These findings are of clinical importance as they encourage the use of this simple and easily available biomarker in the diagnostic approach to patients with abdominal discomfort, especially to decide upon the necessity to perform endoscopy. Patients with fecal calprotectin levels > 50 μg/g should receive either esophagogastroduodenoscopy (EGD) or colonoscopy according to clinical presentation and if negative, follow-up endoscopy, as the prevalence of significant findings with negative initial endoscopy reached 57% in these patients.

Over the last decade, the number of endoscopies performed by gastroenterologists has steadily increased both in the USA and in Europe [28,29]. Given the limited resources and ever increasing health-care costs, optimizing the appropriate selection of patients for endoscopy is crucial. Unfortunately, selection based on symptoms is not reliable; even when compared to an expert panel, individual gastroenterologists tend to overestimate the appropriateness of endoscopies they perform [30]. The Danish Dyspepsia Group found clinical diagnosis of epigastric pain for more than two weeks unreliable and half of all patients with peptic ulcer or reflux esophagitis were misclassified [3]. In another study, neither age nor the presence of alarm features were effective predictors of endoscopic findings in patients with upper abdominal pain [31]. The diagnostic yield of colonoscopy in patients with symptoms other than bleeding or diarrhea was shown to be equal to a screening population [32] and average risk patients with non-specific abdominal symptoms have similar rates of adenomatous polyps as asymptomatic patients [33,34]. Both the *American Society for Gastroenterological Endoscopy (ASGE) *and the *European Panel on the Appropriateness of Gastrointestinal Endoscopy (EPAGE) *have released guidelines in an attempt to optimize patients selection for endoscopy [35-37]. Applying these guidelines yielded significantly more findings for appropriate than for inappropriate endoscopies, but the selection criteria suffered from low specificity [38,39].

Calprotectin is a calcium binding protein of neutrophil granulocytes that correlates well with neutrophil infiltration of the intestinal mucosa when measured in feces, has antimicrobial activity, and is resistant to enzymatic degradation both in vivo and in vitro [9,40]. Accordingly, as a marker of neutrophilic intestinal inflammation, calprotectin values might reflect a composite endpoint for organic intestinal disease. In fact, a mean sensitivity and specificity of 83% and 84%, respectively, has been reported for calprotectin to distinguish organic from non-organic disorders in patients with abdominal discomfort [12]. To separate IBD from non-IBD, the diagnostic accuracy is even higher. Mean sensitivity and specificity was 93% and 96%, respectively, in a recent meta-analysis [11]. Altogether, those studies suggest that elevated fecal calprotectin levels reflect the presence of mucosal inflammation and thus may be used to detect organic disease of the gastrointestinal tract, especially in symptomatic patients.

The value of fecal calprotectin in consecutive patients referred for endoscopy has been poorly studied. In an Italian study, fecal calprotectin was measured in consecutive unselected outpatients undergoing colonoscopy and was found to be elevated in a majority of patients with colorectal cancer and inflammatory conditions of the colon but also in 36% patients with normal endoscopic findings [21]. The negative predictive of fecal calprotectin was 96%.

Our study measured fecal calprotectin in 538 consecutive patients with abdominal discomfort referred for endoscopy. We found that fecal calprotectin was highly useful in the assessment of this important group of patients as it reliably distinguished patients with clinically significant gastrointestinal findings from patients without. Therefore, we confirm findings of prior studies [10-13], but expand the results obtained in these specific patient groups to an unselected group of consecutive patients with abdominal discomfort. It is a remarkable strength of this study that we did not investigate selected patients or patient groups with high clinical suspicion for a specific disorder. In our study, fecal calprotectin provided valuable diagnostic assistance in a heterogeneous patient population. Accordingly, our results favor measurement of fecal calprotectin prior to endoscopic investigation of patients with abdominal discomfort.

Our study further showed that fecal calprotectin had good diagnostic ability also in patients with significant findings of the upper intestinal tract. Previously, elevated fecal calprotectin values have been reported in peptic ulcer disease and gastric cancer [19,20] but the diagnostic value in consecutive patients referred for EGD has never been systematically examined. We found calprotectin to be a valuable biomarker in the evaluation of upper abdominal discomfort; especially when considering the initial clinical approach to these patients is still open to debate. Currently, prompt endoscopy is usually recommended to evaluate new onset dyspepsia for patients over 45 years or with alarm features to rule out malignancy [41], but using these criteria, the high sensitivity to detect relevant findings or new malignancies (82% and 97%) was substantially impaired by low specificity (26% and 20%) [42]. In our study, using fecal calprotectin gave a lower sensitivity (59%) but provided a much higher specificity (84%).

Fecal calprotectin values of patients with esophagitis LA grade A (mucosal breaks < 5 mm, limited to a single mucosal fold) were similar than in patients with normal findings (Table 2). This finding merits special consideration. In the LA system, grading of esophagitis has been limited to erosions in an attempt to increase inter-observer agreement. However, for esophagitis grade A, considerable inter-observer variability has been described (kappa value 0.167) [43]. Endoscopic grading of esophageal erosions is far from perfect, especially for smaller lesions, and this might have contributed to the low sensitivity of fecal calprotectin to detect espohagitis grade A. The impaired sensitivity should be recognized and integrated in the clinical evaluation of patients with abdominal discomfort, especially in the presence of concomitant reflux symptoms.

The diagnostic precision was uniformly high, both in younger patients (age < 50 years) with a low pretest probability, and older patients at higher risk for organic intestinal disease. We also cannot confirm that fecal calprotectin values increase with age in patients with normal findings on endoscopy, as suggested from data in healthy volunteers [44].

All together, those results support the concept that fecal calprotectin is a useful marker in the evaluation of patients with abdominal discomfort and that a biomarker-guided strategy might have additional value to a strategy using clinical decision, including guidelines of appropriateness, to decide on endoscopy. It is important to recognize that these results apply only to symptomatic patients with abdominal discomfort. Especially screening endoscopies such as colonoscopy should be performed irrespective of calprotectin values. Fecal calprotectin has not been established as screening tool for colorectal cancer in asymptomatic patients.

Several limitations of the study merit consideration. First, our prospectively defined endpoint was the presence of a clinically significant finding. Indeed, classification of findings in clinical practice is challenging and endoscopic findings might not always be congruent with abdominal discomfort presented by the patient. Second, in several important gastrointestinal disorders, such as small bowel bacterial overgrowth, celiac disease, or food lactose intolerance, fecal calprotectin levels will be normal [45,46]. Third, we did not systematically assess the presence of mucosal lesions in the small-bowel. We acknowledge, that this is a limitation of our study as increased fecal calprotectin has been shown in small-bowel enteropathy [47]. In the 15 patients who had small bowel capsule endoscopy no significant lesion were found. Fourth, there have been reports of elevated calprotectin values in expectorations of patients with acute and chronic pulmonary disease [48]. This might have influenced fecal measurement of calprotectin.

## Conclusion

In conclusion, our study showed that fecal calprotectin values are elevated in patients with organic gastrointestinal disease. We confirmed results of previous studies showing excellent ability of fecal calprotectin to identify mucosal lesions in the colon. Additionally, we expanded the role of fecal calprotectin as a diagnostic test to the upper intestinal tract by demonstrating its ability to identify esophageal and gastric mucosal lesions. Further prospective studies directly comparing recommended guidelines of appropriateness for endoscopy with fecal calprotectin measurements are warranted to establish the value of a biomarker-guided assessment of patients with abdominal discomfort and to explore the cost-effectiveness of such an approach.

## Competing interests

Christian Niederberger is an employee of Bühlmann Laboratories AG. All other authors report no conflict of interest.

## Authors' contributions

MM, EB and CB participated in study concept and design, acquisition of data, analysis and interpretation of data, drafting of the manuscript, and critical revision of the manuscript for important intellectual content. They also had full access to all of the data in the study and take responsibility for the integrity of the data and the accuracy of the data analysis. CR performed all calprotectin measurements. NT and CN gave important technical advice concerning calprotectin measurement. LR and FSL participated in acquisition of data, analysis and interpretation of data and critical revision of the manuscript for important intellectual content. All authors read and approved the final manuscript.

## Pre-publication history

The pre-publication history for this paper can be accessed here:

http://www.biomedcentral.com/1471-230X/12/5/prepub
